# The Two Visual Systems Hypothesis: New Challenges and Insights from Visual form Agnosic Patient DF

**DOI:** 10.3389/fneur.2014.00255

**Published:** 2014-12-08

**Authors:** Robert L. Whitwell, A. David Milner, Melvyn A. Goodale

**Affiliations:** ^1^Graduate Program in Neuroscience, The University of Western Ontario, London, ON, Canada; ^2^Department of Psychology, The University of Western Ontario, London, ON, Canada; ^3^Brain and Mind Institute, The University of Western Ontario, London, ON, Canada; ^4^Department of Psychology, Durham University, Durham, UK; ^5^Department of Physiology and Pharmacology, The University of Western Ontario, London, ON, Canada

**Keywords:** patient DF, two visual systems hypothesis, grasping, perception and action, dorsal and ventral streams

## Abstract

Patient DF, who developed visual form agnosia following carbon monoxide poisoning, is still able to use vision to adjust the configuration of her grasping hand to the geometry of a goal object. This striking dissociation between perception and action in DF provided a key piece of evidence for the formulation of Goodale and Milner’s Two Visual Systems Hypothesis (TVSH). According to the TVSH, the ventral stream plays a critical role in constructing our visual percepts, whereas the dorsal stream mediates the visual control of action, such as visually guided grasping. In this review, we discuss recent studies of DF that provide new insights into the functional organization of the dorsal and ventral streams. We confirm recent evidence that DF has dorsal as well as ventral brain damage – and that her dorsal-stream lesions and surrounding atrophy have increased in size since her first published brain scan. We argue that the damage to DF’s dorsal stream explains her deficits in directing actions at targets in the periphery. We then focus on DF’s ability to accurately adjust her in-flight hand aperture to changes in the width of goal objects (grip scaling) whose dimensions she cannot explicitly report. An examination of several studies of DF’s grip scaling under natural conditions reveals a modest though significant deficit. Importantly, however, she continues to show a robust dissociation between form vision for perception and form vision-for-action. We also review recent studies that explore the role of online visual feedback and terminal haptic feedback in the programming and control of her grasping. These studies make it clear that DF is no more reliant on visual or haptic feedback than are neurologically intact individuals. In short, we argue that her ability to grasp objects depends on visual feedforward processing carried out by visuomotor networks in her dorsal stream that function in the much the same way as they do in neurologically intact individuals.

Just a few days after her 34th birthday in 1988, a young woman was taking a shower in her newly renovated cottage and was nearly asphyxiated by carbon monoxide from a poorly vented water heater. Although she had passed out from hypoxia, her partner found her before she died and rushed her to hospital. When she emerged from her coma, it was clear that her brain had been badly damaged from lack of oxygen. Her vision was particularly affected. She could no longer recognize common objects by sight or even her husband and friends. In the days and weeks that followed her accident, she showed some improvement, but in the end she was left with a profound visual form agnosia; in other words, she could no longer identify objects on the basis of their shape. Indeed, in later testing, it became apparent that DF (as she is now known in the literature) could not identify even the simplest of geometric figures, although her ability to see colors and visual textures remained relatively intact.

DF’s ability to perceive the form of objects is so compromised that she cannot distinguish a rectangular block of wood from a square one with the same surface area (Figure 1A). Such blocks are often referred to as “Efron” blocks, after the psychologist, Robert Efron, who first devised shapes such as these to test for visual form agnosia (1). DF cannot even manually estimate the widths of the blocks by opening her finger and thumb a matching amount (2, 3). Nevertheless, one aspect of DF’s visually guided behavior with respect to object form has remained remarkably preserved. When she reaches out to pick up one of the Efron blocks, the aperture between her thumb and finger scales in flight to the object’s width (2–7). Similarly, even though DF cannot distinguish perceptually amongst objects on the basis of their orientation and shape, she orients her wrist correctly when posting her hand or a wooden card through a slot (2, 8, 9) and places her fingers on stable grasp points when picking up smooth-spline, pebble-like shapes [Figure 1B; see Ref. (10)]. In other words, despite a profound deficit in form perception, DF seems able to use information about object form to guide her grasping movements.

**Figure 1:**
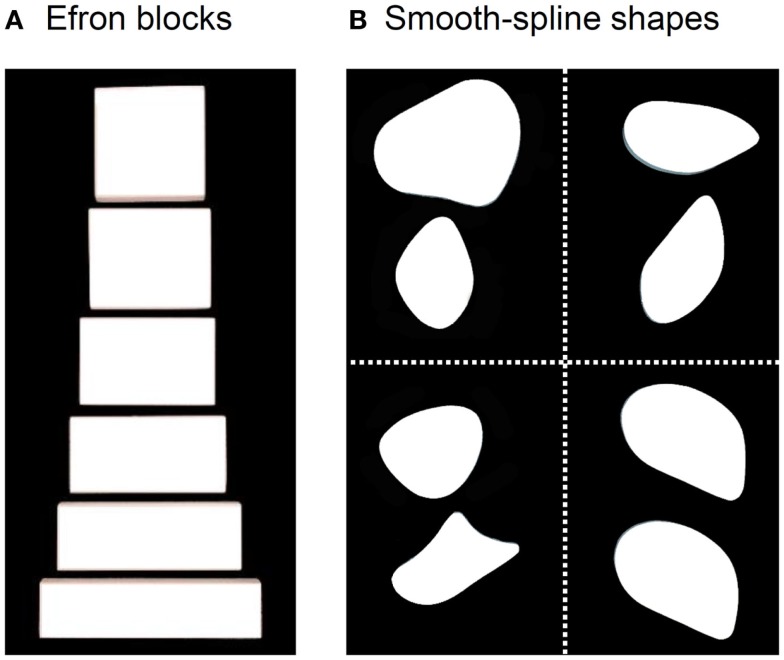
**(A)** Examples from a set of Efron blocks that, by definition, are matched for surface area, texture, mass, and color, but vary in width and length (1). In the grasping task, DF reached out to pick these objects up across their width. In a typical perceptual task, she is asked to indicate manually the width of the block by adjusting her thumb and index-finger a matching amount or to provide same/different judgments about pairs of these objects. **(B)** Examples of the pebble-like shapes used in Goodale et al. (10). DF was asked to either (i) reach out to pick up the shapes presented at one of two possible positions one at a time or (ii) give explicit same/different judgments about pairs of shapes when they had different shapes and different orientations (top left), the same shape but different orientations (top right), different shapes but same orientations (bottom left), and same shape and orientation (bottom right).

DF’s dissociation was one of the key pieces of evidence for the original formulation of the Two Visual Systems Hypothesis (TVSH) put forward by Goodale and Milner in 1992 (11). According to the TVSH, the ventral stream of projections from early visual areas to the inferotemporal cortex mediates vision for perception, whereas the dorsal stream of projections to the posterior parietal cortex mediates the visual control of actions. DF was later shown to have bilateral damage in her ventral stream, particularly in a region of the lateral occipital cortex (area LOC; see Figure 1) implicated in object recognition [for review, see Ref. (12)]. Other patients, who have damage to the dorsal but not the ventral stream, show clear deficits in visuomotor control but relatively spared visual perception (10, 13, 14). Although this double dissociation is by itself compelling, the TVSH is also supported by a broad range of additional evidence extending from monkey neurophysiology to neuroimaging studies of both patients and neurologically intact individuals [for review, see Ref. (15–18)].

Nevertheless, it is important to acknowledge that DF’s lesions are not restricted to her ventral stream. Her brain shows the typical pattern of diffuse atrophy that is seen in patients who have experienced hypoxia from carbon monoxide poisoning, but in her case the cortical thinning is most evident in the posterior regions of the cerebral cortex (see Figure 2). Moreover, in addition to the bilateral damage to LOC in her ventral stream, the original clinical scans also showed evidence of localized damage in the parieto-occipital cortex (POC) of her left hemisphere (2). Subsequent high-resolution MRI scans confirmed the presence of a POC lesion in the left hemisphere while noting extensive bilateral atrophy in the posterior regions of the intraparietal sulcus and in POC of the right hemisphere (19), and the most recent scans indicate that the lesion to POC is now evidently bilateral (20), suggesting that the atrophy has increased in size in these and other areas (see Figure 2). Nevertheless, functional magnetic resonance imaging (fMRI) makes it clear that, despite the lesions to the POC and atrophy in the surrounding tissue, there is robust activation in the anterior intraparietal sulcus of DF’s brain during visually guided grasping [Ref. (19); see Figure 3]. This dorsal-stream area has long been associated with the planning and execution of prehensile movements in both monkeys (21–24) and neurologically intact humans (19, 25–32). Importantly, the activation in DF’s anterior intraparietal cortex occurs despite the fact that she has functionally complete bilateral damage of LOC, suggesting that the computations that mediate her spared visual control of grasping are not dependent on form processing in the ventral stream.

**Figure 2:**
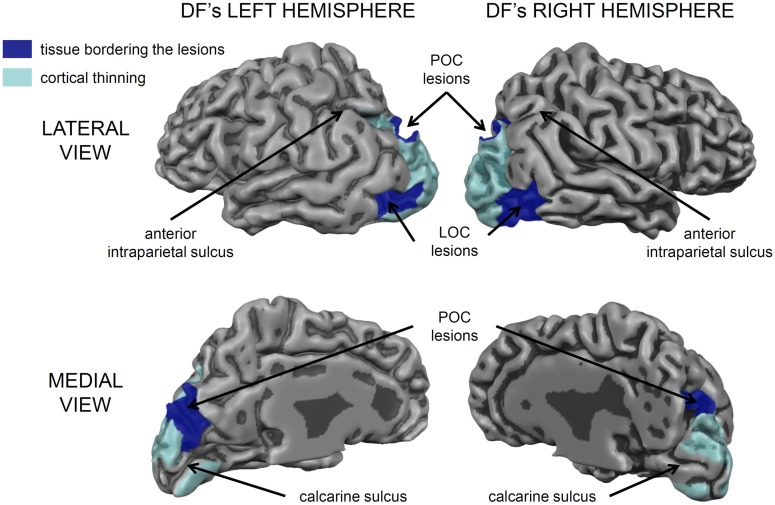
**A 3D rendering of the cortical gray matter boundary of DF’s brain**. The peripheral surface of her gyri is depicted as lighter and more reflective, whereas, the sulci are depicted a darker gray. The areas of cortical thinning are painted in translucent light blue and encompass much of peri- and extrastriate cortex, especially in the left hemisphere [see Ref. (20) for a detailed analysis]. There are also prominent bilateral lesions in the lateral occipital cortex (LOC) and additional lesions in the parieto-occipital cortex (POC) marked in dark blue. Importantly, the cortical tissue surrounding most of the calcarine sulcus, corresponding to primary visual cortex (V1) is intact, as are most of the frontal, temporal, and parietal cortices. The small lesion in the anterior part of the upper bank of the calcarine sulcus in her left hemisphere accounts for the partial quadrantanopia in her lower visual field [see Ref. (10, 33)].

**Figure 3:**
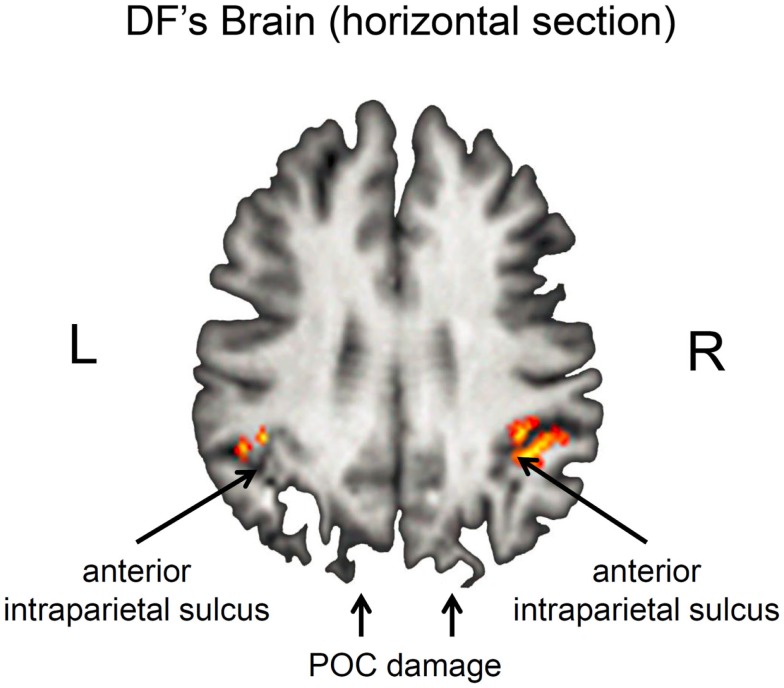
**Horizontal section through DF’s brain illustrating grasp- and reach-related activation in the anterior intraparietal sulcus (aIPS)**. Grasp-specific activation is largely restricted to the right hemisphere. Note that these regions are activated despite the presence of bilateral damage to the parieto-occipital cortex (POC). Unlike healthy controls, there was little or no activation associated with reaching in the POC (19).

The bilateral damage to area POC in DF’s brain warrants some discussion of the role of this brain area, particularly since it forms part of the dorsal stream. After all, the TVSH would predict that damage to this area would affect visually guided action. In fact, a mounting body of evidence implicates POC in the control of visually guided reaching, particularly to targets presented in the periphery [for review see Ref. (34–38)]. In an important study, Karnath and Perenin (38) carried out an analysis of lesion sites in 16 optic ataxic patients with unilateral damage to either the left or the right posterior parietal cortex. The authors contrasted these patients with control patients who had sustained damage to their parietal cortex but who did not exhibit optic ataxia. Their analysis showed that the greatest degree of lesion overlap that was unique to the optic ataxic patients occurred in POC and in the precuneus. Critically, all of the patients with optic ataxia showed misreaching errors when reaching out to touch targets presented in the periphery of their contralesional field. Although there is clear evidence that optic ataxia can include visuomotor deficits in central vision [e.g., Ref. (13, 14, 39, 40)], it is well-known that optic ataxia more frequently manifests itself as misreaching to targets presented in the periphery (41, 42). In fact, peripheral and centrally guided reaches might well rely on separate networks in the posterior parietal cortex (43, 44). Clavagnier et al. (43) have argued that the POC forms part of a fronto-parietal network of areas that is critical for visually guided reaches to peripherally presented targets.

Given the damage to DF’s POC, it is perhaps not surprising that this region shows unusually little, if any, fMRI activation in this region when she reaches out to touch targets (19) and that she exhibits a gross deficit when reaching out to point to targets in the periphery, but not when pointing to targets presented centrally (33, 45). Thus, DF’s deficit in peripheral reaching is likely due to the damage in her POC. There is also some indication that the POC in monkey and in man plays a role in the control of grasps that are directed at peripheral targets (46–48). For example, patient MH, who developed optic ataxia following a unilateral POC lesion, not only shows a deficit in pointing to targets presented in the periphery of his contralesional field, but he also shows a deficit in grip scaling when grasping these same objects. Critically, however, if the objects are closer and he does not have to reach out toward them before picking them up, MH’s grip scaling is normal. This suggests that his grasping deficit is secondary to his deficit in reaching (49). Interestingly, DF also shows a deficit in grip scaling when reaching out to pick up targets located in her peripheral visual field (33). But again, this deficit in grasping targets in the periphery might be secondary to her demonstrated deficit in reaching into the periphery, as it is in patient MH.

Nevertheless, DF’s visuomotor performance, even centrally, is not completely normal in all situations. Himmelbach and colleagues (50) revisited DF’s grasping with the aim of testing for a dissociation using the independent sample *t*-tests recommended by Crawford et al. (51). Himmelbach et al. compared her performance [as reported in Ref. (2, 10)] with that of 20 new age-matched control participants on three different visuomotor tasks: posting a hand-held card through a slot, picking up Efron blocks of varying width, and picking up smooth-spline pebble-like shapes (2, 10). Although DF’s grip scaling (as measured by correlations) with rectangular objects fell within the range of the new control participants, the grasp points she selected when picking up the pebble-like shapes were not as optimal as those of the new control participants tested by Himmelbach et al. Her performance on the card-posting task was also slightly, but significantly, poorer than that of the controls. Nevertheless, as the authors themselves admit, the tests also revealed that DF’s data set satisfied Crawford et al.’s (52) criterion for a “strong/differential” dissociation. Unlike the criterion for a “classic” dissociation in which the patient shows a deficit in one task but not the other, the criterion for a “strong/differential” dissociation allows for a deficit in both tasks, but, critically, requires a dramatically greater deficit in one task than in the other. In other words, despite the presence of slight impairments, DF’s performance on the action tasks were consistently better than her performance on the corresponding perceptual tasks – and this difference was much larger for her than it was for the controls.

Although DF’s spared visuomotor abilities have been examined in a number of different settings, it is her ability to scale her grip aperture to the relevant dimension of a goal object when picking it up that has been tested most often. No matter how the computations underlying the programming and control of grasping are conceptualized [e.g., Ref. (53–59)], there is general agreement that the accurate grasping of a goal object normally requires a visual analysis of the object’s shape so that the final positions of the thumb and fingers can be computed correctly with respect to the relevant dimension of the object, such as its width. Any error in this computation could lead to the object being knocked away or fumbled. When assessing DF’s grasping ability, investigators have typically relied on the known positive linear relationship between the maximum opening of the hand mid-flight and object’s targeted dimension (see Figure 4). Given the survey of DF’s dorsal-stream damage discussed above and in light of Himmelbach’s findings, we examined DF’s grip scaling (as measured by regression slopes) across a range of studies in which she grasped centrally located targets under naturalistic viewing conditions which included online visual feedback (2–7). Critically, the targets in all these studies were drawn from a set of blocks that varied in width and length but were matched for surface area, texture, mass, and color, so that she could not discriminate one from another in perceptual tests. DF clearly scales her grip aperture to the widths of these targets when reaching out to pick them up (see Figure 4). Nevertheless, she does show a modest, though significant, deficit when compared to the controls. Critically, from study to study, DF’s estimations of the widths of these targets remain at chance, whereas, not surprisingly, the estimations made by the controls are essentially perfect. Moreover, a formal test of the difference in performance across the two conditions indicates a significant strong/differential dissociation (52). In short, over the course of two decades of testing, DF’s dissociation between object vision-for-action and object vision for perception remains as strong as ever.

**Figure 4:**
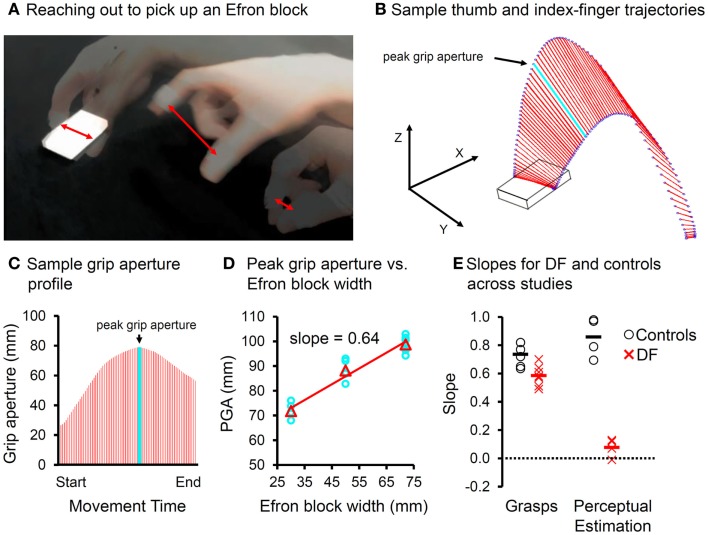
**(A)** Superimposed snapshots of a reach-to-grasp action directed at an Efron block. Red double-headed arrows indicate “grip aperture”, the Euclidean distance between the tracked markers placed on the tips of the thumb and index-finger **(B)** sample trajectories of the thumb and index-finger (blue circles) during a precision pincer grasp as the hand reaches out toward the object. The grip aperture is indicated in red. The light blue line reflects the peak grip aperture, which is achieved well-before the fingers contact the object. **(C)** Grip aperture plotted as a function of time (e.g., percent movement time). The peak grip aperture is again indicated in light blue. **(D)** Peak grip aperture shows a positive linear relationship to the target size of the object, and so it is thought to reflect the visuomotor system’s anticipatory estimate of the target’s width. The slopes can be used as indicators of “grip scaling.” **(E)** The slopes for grasping and manual estimation for both the controls (open circles) and DF (X’s) across studies in which Efron blocks were used, the visual conditions were “ecological” (i.e., online visual feedback was available), and the controls were gender-matched and age-appropriate for DF. Although DF scales her grasp to the width of the Efron blocks, her slopes are significantly shallower than those of the controls, using either independent or paired-samples variants of the *t*-test (*p*_max_ < 0.04). The slopes of DF’s manual estimations are essentially zero and clearly different from those of the controls (*p*_max_ < 6 × 10^−3^). Critically, the difference in slopes between the grasping and manual estimation tasks falls well-outside of the range of the controls (*p*_max_ < 5 × 10^−3^). In other words, across a number of comparable studies of DF’s grasping and perceptual estimation ability, her performance when grasping Efron blocks is sharply dissociated from her performance when perceptually estimating their width.

As remarkable as DF’s visually guided grasping is, however, it is clearly not without limitations. In fact, there are a number of seemingly simple task modifications that have a remarkably detrimental effect on her grip scaling. For example, if a target object is shown to DF and then taken away, she is unable to scale her grasp appropriately when she is asked to show how she would pick the target up should it have remained there. In healthy participants, of course, grip aperture still correlates well with the object’s width, even for delays as long as 30 s. In DF, however, all evidence of grip scaling disappears after a delay of only 2 s (3). DF’s poor performance cannot be due to a general impairment in memory: she has no difficulty showing how she would pick up an imaginary orange or a strawberry, objects that she would have encountered before her accident or would have handled in the past. In other words, when she pretends to pick up an imaginary orange, her hand opens wider than it does for an imaginary strawberry (3). Moreover, she is as accurate as normally sighted controls when asked to open her finger and thumb a particular amount (e.g., “show me how wide 5 cm is”) with her eyes closed. Indeed, her manual estimations in this task are much better than they are when she is asked to indicate the width of an Efron block placed directly in front of her. It is important to note that even though the grasping movements made by normal participants in the delay condition are scaled to the width of the remembered objects, they look very different from those directed at objects that are physically present. This is because the participants are “pantomiming” their grasps in the delay conditions, and are thus relying on a stored perceptual representation of the object they have just seen. Presumably, DF’s failure to scale her grasp after a delay arises from the fact that she cannot use a stored percept of the object to drive a pantomimed grasping movement because she never “perceived” the target object in the first place.

DF’s inability to pantomime grasps becomes relevant in the context of a more recent series of experiments on DF’s grasping abilities, which prompted the suggestion that her ability to grasp objects accurately relies critically on haptic feedback rather than on visual feedforward processing as is the case in normal individuals. Using an ingenious mirror apparatus, Schenk (60) demonstrated that DF’s grip scaling is completely abolished in a task in which the target remains visible (as a virtual image in the mirror) yet is physically absent (behind the mirror) so that when her hand closes down on the apparent edges of the virtual target, it closes down on “thin air.” Schenk argued that DF’s failure to show grip scaling in this situation is due to the absence of haptic feedback, which would compensate for her poor visual abilities. According to Schenk, DF’s grip scaling relies on the integration of visual and haptic feedback about location of the finger and thumb endpoints that are, presumably, applied in a predictive manner on subsequent trials [for a discussion of Schenk’s interpretation and related issues, see Ref. (61, 62)]. When such haptic feedback is absent, Schenk argues, DF’s ability to grasp objects falls apart because her degraded form vision cannot, by itself, support visually guided grasping.

We have offered an alternative, more straightforward explanation. We contend that grasping tasks in which the target is visible but not available to touch are actually pantomime tasks in which the participant has to pretend to contact the object. For the visuomotor systems in the dorsal stream to remain engaged, we would argue, there must be some sort of tactile confirmation that the visible target has been contacted at the end of the movement. In the absence of such feedback, participants revert to pantomiming and pretend to grasp the object they see in the mirror. This conclusion is supported by the fact that the slopes of the function relating grip aperture to object width in the normal participants in the absent-object task are much steeper than those typically observed in normal grasping in which the target object is physically present (63). In fact, the slopes resemble those seen in manual estimations of object width, suggesting that participants are relying on a perceptual representation of the target to drive their behavior rather than engaging more “encapsulated” visuomotor networks in the dorsal stream that normally mediate visually guided grasping. In short, in the absence of any tactile feedback, the participants default to a pantomime grasp. DF, of course, is at an enormous disadvantage in this situation because she does not perceive the form of the virtual image in the mirror and thus cannot generate a pantomimed response. As a consequence, her grip aperture bears no relationship to the width of the target in this situation.

To test this idea, we recently examined DF’s performance using the same mirror set-up used by Schenk (60). In our experiment, however, there was always an object behind the mirror for her to grasp. Importantly, the width of that object never changed, even though the width of the object viewed in the mirror varied from trial to trial (5, 6). With this arrangement, DF always experienced tactile feedback at the end of the movement, but the feedback was completely uninformative about whether or not her grasp was properly tuned to the width of the object in the mirror. Contrary to what Schenk’s visuohaptic calibration hypothesis would predict, we found that DF continued to show excellent grip scaling in this task. In other words, DF was able to use visual information in a feedforward manner to scale her grasp in the complete absence of reliable haptic feedback. Tactile contact by itself was evidently enough to keep the visuomotor systems in her dorsal stream engaged.

It is worth mentioning another prediction that follows from the visuohaptic calibration hypothesis (60, 64). According to Schenk, the reason DF is unable to manually estimate the width of an object is that, unlike in the grasping task, she experiences no haptic feedback about the object’s width after she makes each estimate. We tested this prediction directly by allowing DF to pick up the object immediately after she had made her estimate (6, 7). Again, contrary to the visuohaptic calibration hypothesis, we found that DF continued to be unable to indicate the width of the object despite having accurate haptic information about the width of the target after every estimate. It would appear that an explicit estimate of size, reflecting what she perceived (or perhaps more correctly, did not perceive) of the object’s width, could not take advantage of the haptic feedback.

As we pointed out earlier, the TVSH does not rest entirely on the evidence from DF. Support for the central ideas of the hypothesis comes from a broad range of studies, from monkey neurophysiology to human neuroimaging. Moreover, there is also converging evidence from other patients with visual form agnosia. Patient JS, for example, has bilateral lesions in the ventral stream that were more medial than DF’s, but showed a similar dissociation between visual form perception and the visual control of grasping (65). In fact, there are a number of anecdotal reports in the long literature on visual form agnosia that such patients are able to reach out and grasp objects with surprising accuracy [e.g., Ref. (66)].

Patient DF’s ability to use object form to guide the configuration of her grasping hand in the absence of conscious awareness of that form is reminiscent of what Weiskrantz and his colleagues called “blindsight” in an influential article published in *The Lancet* in 1977 (67). Patients with blindsight are able to respond to visual stimuli presented in their blind field despite a complete absence of visual phenomenology in that field. In fact, subsequent investigations of patients with “action” blind sight [for review, see Ref. (68)] have revealed a dissociation between prehension and perceptual size-estimation (69–73). These patients typically have lesions to the earliest visual cortical areas, including primary visual cortex or even the pathways from the lateral geniculate nucleus that innervate these areas. In a recent paper, Whitwell, Striemer, and Goodale (73) found that a young woman with a unilateral lesion of V1 was nevertheless able to scale her hand to the width of objects that she could not perceive. This observation coupled with many others demonstrating spared visuomotor control in patients with V1 lesions suggests that the posterior parietal cortex enjoys privileged access to visual inputs that bypass the retino-geniculo-striate route. One possible route for such transmission is the well-known set of projections from the superior colliculus in the midbrain to the pulvinar – and from there to the middle temporal area (MT) and the posterior parietal cortex. There are other candidate pathways as well [for review see Ref. (15)]. It seems unlikely that these extra-geniculo-striate projections evolved to be a “back up” should V1 happen to be damaged, but rather play a more integral role in the mediation of visually guided movements in neurologically intact individuals. It seems likely that these pathways normally supply the dorsal stream with essential information for the visual control of movements such as reaching and grasping – and that in DF’s brain such pathways would also be at work.

In summary, the demonstration that DF has a remarkable ability to use information about object form and orientation to control skilled actions despite having a massive deficit in form vision has stood the test of time. Although a number of critics have tried to argue otherwise, it appears that she is able to use feedforward visual information about the shape of objects to guide her hand and fingers as she reaches out to grasp them – and her spared ability to do this does not depend on some sort of abnormal recruitment of haptic information to augment her compromised visual processing. Instead, it appears that vision-for-action in DF, at least as it applies to the control of grasping, depends on the recruitment of relatively intact visuomotor networks in her dorsal stream, and that these networks are engaged in much the same manner as they are in the normal healthy brain.

## Conflict of Interest Statement

The authors declare that the research was conducted in the absence of any commercial or financial relationships that could be construed as a potential conflict of interest.
